# Gut microbiota and metabolic biomarkers in metabolic dysfunction–associated steatotic liver disease

**DOI:** 10.1097/HC9.0000000000000310

**Published:** 2024-02-26

**Authors:** Qichen Long, Fengming Luo, Binghui Li, Ziyang Li, Zhe Guo, Zhiyang Chen, Weimin Wu, Min Hu

**Affiliations:** Department of Laboratory Medicine, The Second Xiangya Hospital, Central South University, Changsha, Hunan, the People’s Republic of China

## Abstract

Metabolic dysfunction–associated steatotic liver disease (MASLD), a replacement of the nomenclature employed for NAFLD, is the most prevalent chronic liver disease worldwide. Despite its high global prevalence, NAFLD is often under-recognized due to the absence of reliable noninvasive biomarkers for diagnosis and staging. Growing evidence suggests that the gut microbiome plays a significant role in the occurrence and progression of NAFLD by causing immune dysregulation and metabolic alterations due to gut dysbiosis. The rapid advancement of sequencing tools and metabolomics has enabled the identification of alterations in microbiome signatures and gut microbiota-derived metabolite profiles in numerous clinical studies related to NAFLD. Overall, these studies have shown a decrease in α-diversity and changes in gut microbiota abundance, characterized by increased levels of Escherichia and Prevotella, and decreased levels of Akkermansia muciniphila and Faecalibacterium in patients with NAFLD. Furthermore, bile acids, short-chain fatty acids, trimethylamine N-oxide, and tryptophan metabolites are believed to be closely associated with the onset and progression of NAFLD. In this review, we provide novel insights into the vital role of gut microbiome in the pathogenesis of NAFLD. Specifically, we summarize the major classes of gut microbiota and metabolic biomarkers in NAFLD, thereby highlighting the links between specific bacterial species and certain gut microbiota-derived metabolites in patients with NAFLD.

## INTRODUCTION

Metabolic dysfunction–associated steatotic liver disease (MASLD) has emerged as a novel, inclusive, and nonstigmatizing nomenclature for NAFLD, which was proposed and officially recognized by a panel of international experts at the 2023 European Association for the Study of the Liver (EASL) conference.^1^ This term falls under the broader category of steatotic liver disease (SLD), which encompasses steatosis resulting from diverse etiologies. The diagnostic criteria for MASLD exhibit some variations from the previously contentious nomenclature of metabolic-associated fatty liver disease.^2^ The diagnostic protocol now requires either imaging or histological evidence of hepatic steatosis, in addition to at least one of the 5 cardiac metabolic risk factors. Furthermore, as per the Delphi consensus, steatohepatitis has been recognized as a vital pathophysiological concept and has been retained, with its designation revised as metabolic dysfunction–associated steatohepatitis.^1^ This decision aims to ensure continued utilization and applicability of data obtained from prior clinical trials and biomarker exploration research based on patients with NASH, while promoting its adoption for individuals now classified as metabolic dysfunction–associated steatohepatitis under the newly endorsed terminology. Whether employing the broad designation of SLD or the more specific MASLD, the proposition of this fresh nomenclature holds the potential to significantly enhance disease awareness and identification. Additionally, this approach enables accurate categorization of fatty liver disease, taking into account the specific etiology of hepatic steatosis. This addresses the longstanding challenge of disease heterogeneity that has previously impeded researchers, thereby fostering drug development and biomarker research. In view of the limited utilization of MASLD in current published studies and its differentiation from NAFLD in terms of disease scope, in this review, we adhere to the corresponding terms as cited in the referenced data.

NAFLD poses a significant public health challenge globally and stands as the foremost cause of liver disease,^3^,^4^ which is estimated to affect about 25% of adults in Western countries,^5^and the prevalence in Asia is 34%,^6^ with an increasing trend. NASH prevalence is projected to increase by 15%–56% in 2016–2030, while NAFLD compensated cirrhosis and end-stage liver disease is estimated to more than double.^7^ In addition to liver disease, NAFLD serves as a risk factor for cardiovascular disease^5^ and kidney damage.^8^ The implications of NAFLD progression are significant; patients with NASH exhibit a more unfavorable prognosis than those with steatosis alone, owing to a heightened likelihood of developing advanced fibrosis and liver-related mortality.^9^,^10^ Nonetheless, NAFLD progression usually transpires asymptomatically; patients generally do not exhibit noteworthy clinical symptoms until they progress to end-stage liver disease. Early detection of hepatic steatosis and accurate assessment of the degree of fibrosis to allow precise intervention in patients at different stages of the disease are essential for the clinical management of patients with NAFLD.

Although biopsy remains the gold standard for the diagnosis and staging of NAFLD, it is unsuitable for mass screening due to its invasive nature and the potential risks of site error, bleeding, abdominal puncture, pneumothorax, etc.^11^,^12^ There is an urgent need to develop noninvasive methods for the diagnosis and staging of NAFLD, which, currently, is achieved by imaging and serum biomarkers. The main imaging techniques are ultrasound, Fibroscan, and MRI. However, they have limitations in the diagnostic accuracy of hepatic steatosis or liver fibrosis, as well as the volatility of results caused by intrahepatic occupant disease.^13^,^14^,^15^,^16^,^17^ Despite having the advantage of being safe, serum markers, such as NAFLD fibrosis score, Fibrosis 4 score, and aspartate aminotransferase to platelet ratio index, have poor predictive value of positivity and can only reflect the degree of liver fibrosis in patients with NAFLD without providing information on the features of hepatic steatosis.^18^ Therefore, the development of robust noninvasive tools for the early diagnosis of NAFLD, as well as for accurate staging, remains a pressing clinical issue.

There is growing evidence that the gut microbiota is strongly associated with the progression of NAFLD.^19^,^20^,^21^ It also has been observed that gut microbiota-derived metabolites play a pivotal role in disease progression.^22^ Considering the indispensable roles gut microbiota and metabolites play in disease progression, methodologies aiming to identify the gut microbiome signature and metabolic profiles of patients with NAFLD may be a promising solution to meet the current clinical demand for accurate staging. 16S rRNA sequencing offers quick identification of the bacterial abundance and changes in microbial community structures. The use of metagenomics enables access to microbial gene and genome composition and pathways, thereby providing a novel aspect of the information on the taxonomic characterization of the microbiome.^23^ In terms of the gut-derived metabolites, untargeted metabolomics has innate advantages in identifying all measurable metabolites in the samples without prior knowledge about the constituents and changes in them, while targeted metabolomics is applicable to absolute quantitative analysis of preselected metabolites. Therefore, developing viable biomarkers based on gut microbiome and metabolic profiles with appropriate methodologies is conducive to accurate diagnosis and staging of NAFLD and monitoring of disease progression.

In this review, new insights regarding the pathogenesis of NAFLD are provided by elucidating the mechanisms by which gut dysbiosis results in immune dysregulation and metabolic alteration, with a focus on gut microbiome characterization identified by the use of targeted 16S rRNA amplicon sequencing and metagenomic next-generation sequencing (mNGS). Additionally, we also discuss the merits and demerits of untargeted and targeted metabolomics in screening and analyzing gut microbial compounds, highlighting bile acids (BAs), short-chain fatty acids (SCFAs), and trimethylamine N-oxide (TMAO) as potential biomarkers for the diagnosis and staging of NAFLD. Further, research progress on microbiome-targeted therapies is summarized to emphasize their potential for the treatment of NAFLD. Notably, based on human studies depicting correlations between the abundance of gut microbiota and metabolite concentrations in patients with NAFLD, we shed light on associations between specific bacterial species and certain gut microbiota-derived metabolites, which may contribute to the exploration of undiscovered biomarkers for diagnostic modeling or validation of latent biomarkers in a larger, prospective cohort.

## MECHANISMS OF GUT MICROBIAL ALTERATION-ASSOCIATED NAFLD

**FIGURE 1:**
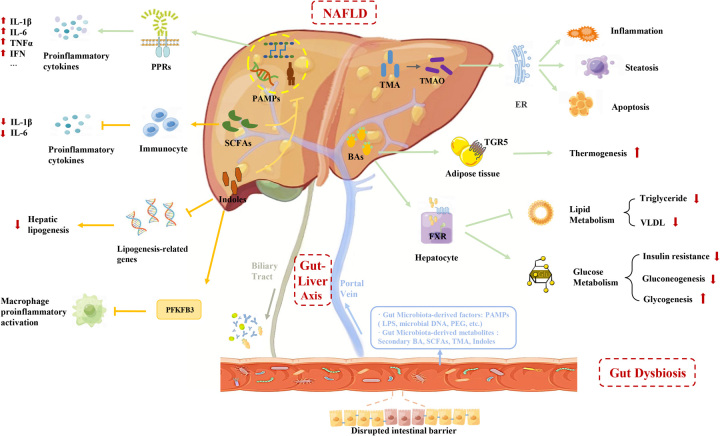
Specific mechanisms of gut microbiota-derived in NAFLD progression. In this figure, the arrows reflect hypothetical relationships, not direct causal links between the pathological mechanisms and NAFLD. The gut-liver axis is an anatomically and functionally closely related structure that allows bidirectional interaction between the gut microbiome and the liver. Here, we only focus on the unidirectional roles that gut microbiota-derived components exert on the liver in NAFLD. Under gut dysbiosis conditions, gut microbiota-derived components translocate into the liver. PAMPs, including LPS, PGN, and microbial DNA, activate the innate immune system and trigger chronic low-grade inflammation by binding to pattern recognition receptors (PPRs), such as TLRs and NLRs. BAs, acting as signaling molecules, on the one hand, could bring about effects of suppressed lipogenesis, reduced gluconeogenesis, and increased insulin sensitivity by activating nuclear receptors; on the other hand, BAs bind to G protein-coupled bile acid receptors in adipose tissue and play a role in maintaining energy metabolic homeostasis. TMA is converted in the liver to TMAO, the latter binds to endoplasmic reticulum stress enzymes and further induces apoptosis and inflammation. SCFAs could inhibit the expression of pro-inflammatory factors and maintain low levels of inflammation. Indoles can not only induce the upregulation of PFKFB3, thereby suppressing the inflammatory response, but also reduce the expression of numerous lipogenesis-related genes, such as Srebf1, ACCA1, PPARγ, etc. In addition, both indoles and SCFAs contribute to restraining the entry of LPS into the circulation and the liver by strengthening the intestinal barrier. The processes that promote NAFLD progression are represented in green arrows and lines, and the ones that hamper NAFLD progression are represented in yellow. Abbreviations: ACCA1, acetyl-CoA carboxylase1; BA, Bile acid; FXR, farnesoid X receptor; IFN, interferon; LPS, lipopolysaccharide; NLR, nod-like receptors; PAMP, pathogen-associated molecular patterns; PGN, peptidoglycan; PPARγ, peroxisome proliferator-activated receptors; SCFA, short-chain fatty acids; Srebf1, sterol regulatory element-binding protein1; TGR5, bile acid G protein-coupled receptor 5; TLR, toll-like receptors; TMA, Trimethylamine; TMAO, trimethylamine oxide.

The gut microbiota refers to the collection of bacteria, archaea, viruses, and eukaryotic microorganisms present in the human gastrointestinal tract. These microorganisms exchange signaling molecules and substrates with the human host, thereby regulating the cellular physiology and immune response of the host and impacting their overall health state.^24^,^25^ The liver is primarily connected to the intestinal tract through the portal vein, and the gut-liver axis is the main mechanism by which the gut microbiota and liver interact. On the one hand, bioactive substances produced in the liver, such as BAs and antimicrobial molecules, can enter the intestine to regulate the growth of flora. On the other hand, metabolites of the gut microbiota can enter the liver with the portal vein circulation to regulate the liver’s function.^26^ Under normal conditions, only a small percentage of gut microbiota-derived components enter the liver due to the presence of the intestinal barrier; in patients with NAFLD, gut dysbiosis causes the intestinal epithelial barrier disruption, leading to the translocation of a large number of gut microbiota-derived components to the liver through the portal vein.^27^,^28^ These components cause hepatic metabolic alterations and a series of inflammatory reactions. These components cause hepatic metabolic alterations and a series of inflammatory reactions. Moreover, the altered components of bioactive substances secreted by the dysfunctional liver prevent the restoration of the intestinal ecosystem, leading to a vicious cycle that promotes the progression of NAFLD.^29^ The deleterious impacts of gut microbiota-derived components on NAFLD are intricately linked to immune dysregulation and metabolic alterations, which are illustrated in Figure 1.

### Gut microbial alteration and the immune system

Gut microbiota-derived factors, namely pathogen-associated molecular patterns (PAMPs), include lipopolysaccharide (LPS), peptidoglycan, microbial DNA, etc. PAMPs are generally believed to be associated with NAFLD progression by triggering pattern recognition receptor signaling, which leads to hepatic steatosis, insulin resistance, and activation and recruitment of inflammatory cells.^30^ LPS, an endotoxin present in the cell wall of gram-negative bacteria, could translocate to the liver by bacterial extracellular vesicles and binds to toll-like receptors, activating hepatic immune cells and leading to the release of pro-inflammatory cytokines such as TNFα, IL-6 and IL-1β, which initiate and maintain the inflammatory cascade response, ultimately leading to liver fibrosis.^29^ Peptidoglycan is regarded as another potent PAMP that could result in the progression of steatosis and inflammation in liver by activating NOD2, one of the nod-like receptor family.^31^ Bacterial DNA can also aggravate liver inflammation by mediating the release of pro-inflammatory factors and inducing programmed cell death.^32^ In addition to PAMPs, some gut microbiota-derived metabolites, such as SCFA and tryptophan metabolites also regulate the immune status. The activation of various immune cells, such as macrophages, Natural Killer T cells, and γδ T cells, is closely related to the gut microbiota.^33^ And low levels of inflammation maintained by gut microbes and their metabolites can also drive the development of obesity and insulin resistance. Obesity and insulin resistance, as high-risk factors for NAFLD, promote the development of NAFLD.^34^

### Gut microbial alteration systemic metabolism

Gut microbial alteration can also affect systemic metabolism. The production of SCFA and the conversion of primary BAs to BAs cannot be achieved without the involvement of the gut microbiota, which are important in regulating glucose metabolism and maintaining energy metabolism balance.^35^ What’s more, recent studies have shown that gut microbes are also able to influence NAFLD by affecting iron metabolism. Serum ferritin levels are positively correlated with hepatic lipid accumulation. Mayneris-Perxachs et al^36^ found that ferritin was negatively correlated with Pasteurellaceae, Leuconostocaceae, and Micrococcaea families.

Collectively, translocation of bacteria/bacterial metabolites to the liver leads to disruption of immune function and systemic metabolism in the body, and may even be trapped in a vicious cycle, which further aggravates the disease. Monitoring alterations in the composition and abundance of gut microbiota and their metabolites is anticipated to provide new insights into the diagnosis and treatment of NAFLD.

## GUT MICROBIOTA CHARACTERIZATION OF NAFLD

### Next-Generation Sequencing methods to assess gut microbiota

**FIGURE 2:**
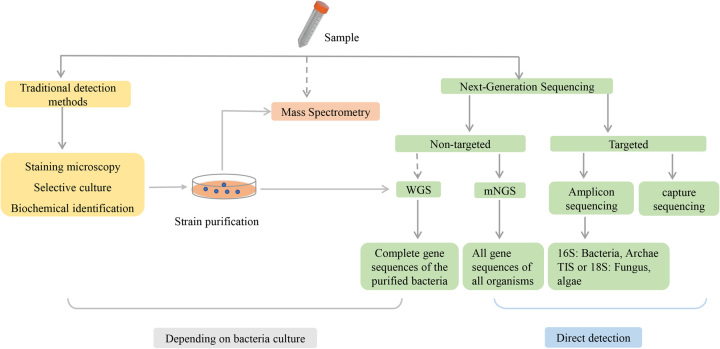
Common methods for microbial identification.

To investigate the impact of gut microbiota on NAFLD, one approach is to analyze variations in the microbiota composition, as illustrated in Figure 2. However, the diversity and vastness of the bacterial population in the human intestine, estimated to be around 1014 bacteria, make it challenging to comprehensively understand the changes in the gut microbiota composition using conventional culture or mass spectrometry methods.^37^ Next-generation sequencing provides a high-throughput approach to reveal changes in the structure of gut microbiota. Next-generation sequencing can be categorized into targeted and nontargeted methods. Targeted sequencing involves the detection of specific sequences (target sequence) in the gene through targeted amplification and detection. Targeted 16S rRNA amplicon sequencing is commonly employed for detecting bacteria, while 18S rRNA sequencing is used for detecting fungi and ITS. Another targeted method is to use capture probes to hybridize with macrogenomic libraries for enrichment purposes and increase the detection rate of target microorganisms. Nontargeted sequencing can be performed using whole genome sequencing or mNGS. Whole genome sequencing accurately distinguishes genetic differences between strains but is time-consuming and laborious, as it requires the isolation of pure colonies from specimens after culture. In contrast, mNGS directly tests the specimens sent, obtaining all genetic information.^38^ Targeted 16S rRNA amplicon sequencing and mNGS are most commonly used in clinical studies of intestinal microorganisms.

#### Targeted 16S rRNA amplicon sequencing

The 16S rRNA gene is ubiquitous in bacteria, so the relationship between almost all bacteria can be identified by 16S rRNA sequencing. The gene sequence is ~1550 bp long and consists of variable regions (V1-V9) and conserved regions.^39^,^40^ 16S rRNA sequencing has been widely used to characterize bacterial communities, which uses the Illumina sequencing platform to target, amplify, and sequence portions of the highly variable region.^41^ In general, 16S rRNA sequencing can identify microorganisms to the phylum or genus level but may be less accurate at the species level because short sequence sequencing may not provide sufficient sequence differences.^42^ With the development of PacBio and Oxford Nanopore sequencing platforms, full-length 16S rRNA sequencing becomes possible. Full-length 16S rRNA sequencing is accurate enough to distinguish single nucleotide substitutions between 16S genes, and proper processing is expected to differentiate microorganisms at the species and strain levels.^40^

#### Metagenomic next-generation sequencing (mNGS)

Contrasted with 16S rRNA sequencing, mNGS has a higher resolution, and can identify a more diverse bacterial species.^43^ mNGS detects all genes present in all organisms of testing samples rather than targeting specific gene sequences.^44^ Since mNGS can capture all gene sequences, it can also identify fungi and viruses present in the sample, which cannot be identified by 16S rRNA sequencing.^44^ However, mNGS also has several limitations; the detection of microbial DNA can be interfered by human DNA in the sample, in addition to the higher cost and longer sequencing time that need to be considered when choosing mNGS.^45^

In current research, 16S rRNA sequencing has been the preferred method for analyzing changes in microbial diversity and identifying disease-associated flora. However, relying solely on 16S rRNA amplicon sequencing to investigate the relationship between microorganisms and diseases may result in the loss of important information. The alterations in microbial populations at the genus level differ from those at the species level. Hence, investigations into microbial species using mNGS are more likely to reveal changes in population function and enable the identification of more reliable markers.^46^

### Gut microbiota in NAFLD

In general, the majority of bacteria in the healthy human intestine are classified into the Firmicutes and Bacteroidetes phyla, while Proteobacteria, Actinobacteria, Fusobacteria, and Verrucomicrobia phyla are relatively rare. Among the Bacteroidetes, 2 dominant genera, Bacteroides and Prevotella, can be found, whereas the dominant genera of Firmicutes are Ruminococcus, Blautia, Eubacterium, and Faecalibacterium.^47^ The diversity of gut microbiota plays an important role in maintaining human health, and lower α-diversity is generally associated with inflammation, obesity, and insulin resistance.^48^ A study conducted on 107 adolescents revealed a decreasing trend in α-diversity with increasing levels of liver fat.^49^ Astbury et al^48^ described microbiome differences in 65 patients with biopsy-proven NASH, and confirmed the previous findings that α-diversity was lower in patients with NASH and further decreased in patients with cirrhosis. Characterizing specific patterns of change in gut microbiota composition for potential biomarkers has been a hot topic in the field. A meta-analysis revealed that increased Escherichia, Prevotella, and Streptococcus levels and decreased Coprococcus, Faecalibacterium, and Ruminococcus levels were the universal intestinal bacterial signature of NAFLD.^50^ Changes in gut microbiota composition in patients with NAFLD can occur at the phylum, family, genus, and species levels. In this review, we will discuss recent studies on several commonly studied gut microbiota in the development of NAFLD.

#### Faecalibacterium

Faecalibacterium belongs to Firmicutes phyla. Faecalibacterium is the most important butyrate-producing bacterium in the intestine and has been considered an indicator of human health, and the decrease in Faecalibacterium is often associated with inflammation. A significant decrease in Faecalibacterium has been reported in a variety of diseases, for instance, ulcerative colitis, inflammatory bowel diseases, and Type 2 diabetes.^51^

Several recent studies have also reported a decrease of Faecalibacterium in patients with NAFLD . A significant reduction in F. prausnitzii was observed in participants with high hepatic fat content compared to participants with low-fat content.^52^ In a large population study, excluding the effects of obesity and gender, patients with NAFLD still had a significant reduction in Faecalibacterium compared to patients with no-NAFLD .^53^ In mouse studies, it has been demonstrated that F. prausnitzii treatment reduces hepatic fat content and fibrosis in mice.^54^ The mechanisms by which Faecalibacterium improves NAFLD remain unclear. However, some studies have reported possible reasons, and it is observed that F. prausnitzii-derived microbial anti-inflammatory factors upregulate the expression of ZO-1, a key tight junction protein in cell adhesion.^55^ A recent in vitro study demonstrated that F. prausnitzii could recover the impairment of epithelial barrier function caused by inflammatory cytokines and LPS.^56^

#### Akkermansia muciniphila

*Akkermansia muciniphila* (*A. muciniphila*), the only member of Verrucomicrobia phyla, breaks down intestinal mucin as the sole source of carbon, nitrogen, and energy and produces acetate and propionate.^57^ *A. muciniphila* is an abundant resident in the human intestine, accounting for more than 1% of total fecal cells.^58^ Since its first discovery in 2004, numerous studies have confirmed the beneficial role of *A. muciniphila* in a variety of metabolic diseases, including obesity, type 2 diabetes, cardiovascular disease, and NAFLD.^59^,^60^ *A. muciniphila* has been widely considered as a novel promising ‘next-generation beneficial microbe’ for metabolic disease management.^59^

Recent studies have shown that *A. muciniphila* is reduced in NAFLD.^61^,^62^ *A. muciniphila* is associated with the expression of genes related to BA synthesis, metabolism, and transport, which contribute to the maintenance of proper bile formation.^63^ What is more, *A. muciniphila* can eliminate hepatic steatosis by enhancing the oxidation of accumulated lipids.^64^ Several mouse studies have confirmed that *A. muciniphila* can alleviate NAFLD. A recent study indicated that *A. muciniphila* treatment reduced the levels of serum triglyceride, alanine aminotransferase (ALT), and inflammatory cytokine IL-6 in obese mice.^65^ Nishiyama et al^66^ also verified that an increase of *A. muciniphila* has a beneficial effect on hepatic steatosis and liver injury in ob/ob mice. Intriguingly, Rao et al^64^ found that *A. muciniphila* treatment maintained its anti-NAFLD effect after 4 weeks of drug withdrawal, which may facilitate the future clinical application of *A. muciniphila*.

#### Prevotella

Prevotella is one of the dominant genera of Bacteroidetes, and the levels of Prevotella were usually anticorrelated with Bacteroides.^67^ Prevotella is generally considered to be a beneficial bacterium with potential benefits in polysaccharide degradation and SCFA metabolism, maintaining glucose homeostasis.^68^,^69^ However, some studies have also suggested that some Prevotella strains may be pathogenic bacteria and are associated with a variety of chronic inflammatory diseases.^47^,^70^ Whether *Prevotella* has a positive or negative effect may be related to the strain level diversity, namely, *P. copri*, and has a high genetic diversity because of inter-species and intra-species variation.

Prevotella also showed inconsistent results in surveys of the gut microbiome in NAFLD patients. Michail et al^71^ revealed that children with NAFLD had more abundant Prevotella. While in an adult study, the opposite result was obtained; there was a lower abundance of Prevotella in the NAFLD.^72^ Another study conducted in Thais also showed that the Prevotella genus is abundant in subjects with NASH.^73^ These results suggest that additional studies are needed to confirm the changes of Prevotella abundance in NAFLD.

#### Escherichia

Proteobacteria phyla includes many pathogenic bacteria, and Escherichia is also a member of the Proteobacteria phyla. Escherichia is the main pathogenic bacterium producing highly active LPS in patients with fatty liver.^74^ Overgrowth of Escherichia may increase intestinal permeability and LPS levels in the portal vein, which may activate inflammasome and lead to liver injury.^50^ Carpino et al found that serum and hepatocyte Escherichia coli LPS levels were higher in both patients with NASH and NASH mice than in controls, and the elevated LPS could induce liver injury by activating Toll-like receptor 4 positive macrophage and platelet.^44^ Most previous studies have confirmed an increase in Escherichia at the genus level and possibly progressively in the progress of NAFLD to NASH.^75^,^76^ Bacteria of the same genus may possess different pathogenic abilities at the strain level. Recently Zhang et al^77^ identified E. coli NF73-1 as a key strain triggering the progression of NAFLD using whole genome sequencing. Translocation of E. coli NF73-1 to the liver leads to an increase in hepatic M1 macrophages and, ultimately, to a disruption of hepatic lipid metabolism.

#### Ruminococcus gnavus

Ruminococcus is one of the dominant members of Firmicutes. There is some inconsistency regarding the alterations in the abundance of Ruminococcus in NAFLD. Boursier et al^78^ showed that Ruminococcus is increased in patients with NAFLD and correlated positively with the degree of liver fibrosis. However, in another study, Ruminococcus abundance was lower in patients with NAFLD than in healthy controls.^79^ One reason for this may be related to the fact that the Ruminococcus genus contains many different species of strains, which may produce different kinds of metabolites and have different effects on the disease.

Ruminococcus gnavus (R. gnavus), is a bacterium species strongly associated with active inflammatory bowel disease. *R. gnavus* can consume mucin and directly disrupt the intestinal barrier. In addition, it produces an inflammatory polysaccharide that induces the release of TNFα in dendritic cells, exacerbating the inflammatory response.^80^ Using 16S ribosomal RNA gene sequencing, Alferink et al^81^ confirmed the association of R. gnavus with steatosis in 472 patients with steatosis. After adjusting for confounding factors such as IBM and diet, *R. gnavus* remained independently associated with steatosis.

### Gut microbiota alterations in the progression of NAFLD

In recent years, numerous studies have investigated the gut microbiota composition during different stages of NAFLD. Hoyles et al^82^ investigated the correlation between gut microbial diversity and liver steatosis after adjusting for body mass index, which showed that Proteobacteria, Actinobacteria, and Verrucomicrobia were significantly correlated with liver steatosis, while Firmicutes and Euryarchaeota were significantly anticorrelated. Lanthier et al^83^ used 16S rRNA amplicon sequencing to detect a group of samples of patients with NAFLD with different degrees of steatosis and fibrosis evaluated by fiberscan by comparing patients with nonsevere liver steatosis (n=10), severe liver steatosis (n=18), and both severe steatosis and fibrosis (n=9), and they found that Clostridium sensu stricto abundance significantly decreased with the onset of fat deposition and fibrosis. Also, further linear discriminant analysis effect size showed that Escherichia/Shigella may be the discriminating microorganism for fibrosis. A study by Shen et al^72^ also confirmed the presence of Escherichia in patients with significant fibrosis compared to F0/F1 fibrosis. Loomba et al^84^ applied a macrogenomic sequencing platform to distinguish 86 patients with biopsy-proven NAFLD with different degrees of fibrosis. They also found that as mild/moderate NAFLD progressed to advanced fibrosis, the abundance of the Proteobacteria phylum increased while the Firmicutes phylum decreased. They selected 37 bacterial species, alpha diversity, age, and body mass index to construct a random forest model, which can diagnose advanced fibrosis with an AUC of 0.936.

The development of cirrhosis and represents severe outcomes of advanced NAFLD progression. Consequently, the precise identification of individuals with the highest risk of advanced NAFLD is crucial. Oh et al^85^ performed a macrogenomic analysis of the gut microbiota in 54 non-NAFLD controls and 27 NAFLD-cirrhosis, and they identified a number of species, including Veillonella parvula, Ruminococcus gnavus, Faecalibacterium prausnitzii, and 19 other species, whose variation can accurately diagnose AFLD-cirrhosis (AUC 0.91). Ponziani et al^86^ explored the microbial features associated with the progression of NAFLD-related cirrhosis to HCC. Patients with NAFLD-related cirrhosis and HCC (n=21), NAFLD-related cirrhosis without HCC (n=20), and healthy controls (n=20) were studied. The NAFLD-related cirrhosis and HCC groups exhibited a higher abundance of Bacteroides and Ruminococcaceae in comparison to the group without HCC. A summary of gut microbiota alterations associated with NAFLD is presented in Table 1.

**TABLE 1:** Gut microbiota alterations in NAFLD

References (Y)	Participants (n)	Diagnostic tool	Alterations	Region	Analysis method
Leung et al^87^	Non-NAFLD(n = 90) NAFLD(n = 90)	ultrasonography	Decreased methanobrevibacter and phascolarctobacterium, increased Slackia and Dorea formicigenerans could be signatures of NAFLD risk	China	Shotgun metagenomic sequencing
Rau et al^88^	HCs (n = 27) NAFL(n = 14) NASH(n = 18)	Liver biopsy	Fusobacteria and Fusobacteriaceae abundance were increased in patients with NASH	Germany	16S rRNA
Astbury et al^48^	NASH (n = 65) ; healthy controls (n = 76)	Liver biopsy	Increased Collinsella was most strongly associated with NASH	UK	16S rRNA
Iino et al^53^	NAFLD (205) Non-NAFLD (669)	Ultrasonography	Ruminococcaceae and Faecalibacterium were significantly decreased in patients with NAFLD	Japan	16S rRNA
Boursier et al^78^	F0/1 fibrosis (30); F ≥ 2 fibrosis (27)	Liver biopsy	Increased abundance of Bacteroides are independently associated with NASH and increased Ruminococcus with fibrosis.	France	16S rRNA
Alferink et al^81^	no steatosis (883) Steatosis (472)	Ultrasonography	In patients with steatosis, Coprococcus decreased and Ruminococcus Gnavus increased	Netherlands	16S rRNA
Loomba et al^84^	stage 0–2 NAFLD (72) stage 3–4 Advanced Fibrosis (14)	Liver biopsy	From mild/moderate NAFLD to advanced fibrosis, the Proteobacteria phylum increased while the Firmicutes phylum decreased.	America	metagenomic sequencing
Oh et al^85^	non-NAFLD (54) NAFLD-cirrhosis (27)	Liver biopsy MRI-PDFF MRE	Veillonella parvula, Veillonella atypica, Ruminococcus gnavus, Clostridium bolteae and Acidaminococcus sp. D21 was increased, and Eubacterium eligens, Eubacterium rectale, and Faecalibacterium prausnitzii were decreased	America	Shotgun metagenomic sequencing
Da Silva et al^79^	15 simple steatosis (15); NASH (24); healthy controls (28)	Liver biopsy	Ruminococcus, Faecalibacterium prausnitzii and Coprococcus were decreased in patients with NAFLD, an no difference was found between NASH and simple statosis.	Canada	16S rRNA
Lanthier et al^83^	nonsevere liver steatosis (10); severe liver steatosis (18); both severe steatosis and fibrosis (9)	Fibroscan	Clostridium sensu stricto was decreased in patients with severe steatosis, and an enrichment of Escherichia/Shigella is more represented in the gut microbiota from subjects with fibrosis	Belgium	16S rRNA
Hoyles et al.^82^	no liver steatosis (10); liver steatosis 1 (22); liver steatosis 2 (14); liver steatosis 3 (10)	Liver biopsy	Proteobacteria, Actinobacteria and Verrucomicrobia were significantly correlated with liver steatosis, while Firmicutes and Euryarchaeota were significantly anticorrelated.	Italy and Spain	Shotgun metagenomic sequencing
Behary et al^89^	NAFLD-HCC (32); NAFLD-cirrhosis (28); NAFLD control (30)	Transient elastography scores	B. caecimuris and V. parvula are increased only in NAFLD-HCC patients.	Australia	Shotgun metagenomic sequencing
Caussy et al^90^	Non-NAFLD controls(n = 51); NAFLD without advanced fibrosis (n = 17); NAFLD-cirrhosis (n = 25)	MRI-PDFF MRE Fibroscan	Megasphaera was only enriched in the NAFLD-cirrhosis group, and more Gram-negative microbes were observed in advanced fibrosis stage	America	16S rRNA
Ponziani et al.^86^	NAFLD-related cirrhosis and HCC (21); NAFLD-related cirrhosis without HCC (20); healthy controls (20)	—	Bacteroides and Ruminococcaceae were higher in NAFLD-related cirrhosis and HCC	Italy	16S rRNA

Abbreviations: MRI-PDFF, MRI-proton density fat fraction; MRE, Magnetic resonance elastography.

As mentioned above, gut microbiota composition changed during NAFLD progression. Loomba et al^84^ and Oh et al^85^ also suggested that the variation of bacterial species can accurately predict disease progression. However, inconsistent changes in gut microbiota have been reported in patients with NAFLD. Possible explanations for this inconsistency include small sample sizes and the absence of a completely healthy control group in some studies, variations in diagnostic tools for fatty liver, and differences in the composition of the gut microbiota due to different sequencing methods. Therefore, it is important to conduct large-scale, well-characterized studies of MASLD patients using macrogenomic analysis to assess changes in the gut microbiota profile.

## GUT METABOLOMIC CHARACTERIZATION OF NAFLD

### The MS-based metabolomics to detect microbiota-derived metabolite

The microbiota-derived metabolites play an important role in microbial effects on the gut-liver interactions, which can activate or inhibit signaling pathways related to the NAFLD progression. The microbiota-derived metabolites involved in these interactions, including BAs, SCFAs, trimethylamine (TMA) metabolites, tryptophan metabolites, etc., have enormous chemical diversity and a large dynamic range, which bring huge analytical challenges for separation and quantification, especially in biological samples.^91^ Metabolomics provides a comprehensive biochemical analytical technique to characterize and detect all low molecular weight compounds (<1500 Da), which can assess metabolism and identify downstream functions of genes and proteins.^92^,^93^ The metabolomics analysis is primarily achieved using the mass spectrometry technology platform due to its high sensitivity and specificity, also called mass spectrometry (MS)-based metabolomics.^94^ The MS-based metabolomics approach can quantify thousands of metabolites down to pico- and nanomolar levels in a complex biological matrix.^95^ With the MS-based metabolomics approach, microbiota-derived metabolite detection is expected to become a new noninvasive diagnostic strategy for NAFLD. The MS-based metabolomics approach can be classified as nontargeted or targeted, which can be applied in different situations.

#### Targeted metabolomics in NAFLD

Targeted metabolomics are commonly used when the investigators already have prior knowledge about the specific metabolites and require identification confidence and precise quantification of the target metabolites.^96^ Since targeting specific molecules can directly tune the workflow and instrument for the detection of those specific molecules, targeted metabolomics have advantages in high sensitivity, high specificity, and wide linear range, which is considered the best approach for metabolites absolute quantitative analysis.^93^ The analytical methods commonly applied in targeted metabolomics are the target liquid chromatography-tandem mass spectrometry and gas chromatography-tandem mass spectrometry, with hybrid quadrupole-linear ion trap or triple quadrupole mass spectrometers as their MS analyzers.^93^,^97^,^98^ The targeted liquid chromatography-tandem mass spectrometry is generally used in the quantification of polar metabolites (ie, BAs), while target gas chromatography-tandem mass spectrometry used in nonpolar or volatile metabolites (ie, SCFAs). Targeted metabolomics is suitable for clinical application, especially for the target metabolites biomarker validation in large-scale cohorts. However, targeted metabolomics only can quantify the preselected compounds, which limits the potential for the discovery of unexpected metabolite biomarkers.

#### Untargeted metabolomics in NAFLD

Untargeted metabolomics is commonly used to identify all measurable metabolites in the sample without prior knowledge about the constituents and changes in them. As numerous metabolic features need to be identified and analyzed in the untargeted metabolomics, the analytical methods must have excellent resolution, high sensitivity, and a wide dynamic range, and the MS should be equipped with high-resolution MS analyzers, including time of flight, Orbitrap, or Fourier transform ion cyclotron resonance.^91^,^99^ The untargeted liquid chromatography-tandem coupled with high-resolution MS is the major analytical method of untargeted metabolomics, as it has more detectable metabolites with higher sensitivity due to the liquid chromatography-tandem separation compared to direct infusion (shotgun) MS.^93^ With untargeted metabolomics, the similarities and differences of metabolites between different study cohort can be statistically analyzed to discover new biomarker of a particular disease and identify the metabolic pathways related to the disease.

The untargeted metabolomics provides a powerful discovery tool for clinically relevant microbiota-derived metabolite biomarkers for NAFLD. However, it still has its limitations. First, the compound identification can be not good enough limited to the data acquisition speeds, since too large amount of data should be detected. Second, the quantification of metabolites can be inaccurate due to the sensitivity and the interference of matrix effects.^100^ Therefore, the discovered metabolite biomarkers should be validated in the large-scale validation cohort combined with the precise targeted metabolomics method to ensure the effectiveness of the biomarkers for MASLD.

### Gut metabolomics in NAFLD

Gut microbiota is essential in maintaining host health, and variations in the composition of the gut microbiota and metabolites can affect host systemic metabolism.^101^ In a study, Hu et al^102^ performed a comprehensive serum metabolomic analysis of 112 patients with early NAFLD and 112 healthy volunteers by ultra-performance liquid chromatography-Orbitrap mass spectrometry. They identified 55 different serum metabolites, with 15 being closely associated with early NAFLD, and found no significant differences in liver chemistry, including aspartate aminotransferase and ALT. Thus, comprehensive serum metabolomics analysis could potentially unveil novel noninvasive detection markers for patients. Furthermore, Liu et al^103^ suggested that serum untargeted metabolomics may provide a diagnostic model to distinguish subtypes of NAFLD, regardless of Lean-NAFLD or Obese-NAFLD type. Table 2 summarizes several classes of gut microbiota metabolites that have been widely reported in the literature to be involved in the development of NAFLD, including bile acids, SCFA, trimethylamine, and Tryptophan metabolites. Monitoring changes in the composition and levels of these classes of gut microbiota metabolites is important for the diagnosis and staging of NAFLD and is expected to provide new insights into disease diagnosis and treatment.

**TABLE 2:** Gut metabolomic alterations in NAFLD

References (Y)	Participants (n)	Diagnostic tool	Alterations	Region	Analysis method
Yang et al^104^	HCs(n = 30) NAFLD(n = 32)	FibroScan-CAP Ultrasound CT MRI biopsy	Compared with controls, there were 22 differential metabolites shared in feces and serum (including purines and purine derivatives, amino acids, peptides, BAs, derivatives, etc), dominated by lipid molecules	China	Nontargeted metabolomic detection
León et al^105^	Non-NAFL (n = 38) NAFLD (n = 319) NAFL (n = 88) borderline NASH (n = 122) NASH (n = 109)	Biopsy	TMAO and choline levels were significantly increased in NAFLD and NASH; total secondary bile acids concentrations were at a notably higher level in NASH than non-NASH subjects	Mexico	Targeted metabolomic detection (TMAO, choline, betaine, and BAs)
Leung et al^87^	Non-NAFLD (n = 90) NAFLD (n = 90)	Ultrasonography	15 metabolites were significantly different between case and controls, such as 3-chlorotyrosine and phenylacetic acid	China	Nontargeted metabolomic detection
Chen et al^106^	HCs (n = 15) NAFLD (n = 72) non-NASH (n = 25) NASH (n = 22)	Biopsy	The ratio of conjugated chenodeoxycholic acids to muricholic acids was positively associated with the severity of liver lesions	China	Targeted metabolomic detection (BAs)
Nimer et al^107^	HCs (n = 50) NAFLD (n = 102)	Biopsy	Glycine-conjugated forms of the BAs were significant associations with more severe inflammation and fibrosis	America	Targeted metabolomic detection (BAs)
Puri et al^108^	HCs (n = 24) NAFL (n = 25) NASH (n = 37)	Biopsy	In patients with NASH, total primary BAs increased, and secondary BAs decreased	America	Targeted metabolomic detection (BAs)
Rau et al^88^	HCs (n = 27) NAFL (n = 14) NASH (n = 18)	Biopsy	In patients with NAFLD, fecal acetate and propionate levels increased	Germany	Targeted metabolomic detection (SCFAs)
Lee G et al^109^	No-NAFLD (n = 31) NAFLD (n = 171)	Biopsy	In nonobese subjects with NAFLD (BMI＜25 g/m^2^ ), fecal propionic acid level and the synthesis of bile acid increased with worsening fibrosis severity	Korea	Targeted metabolomic detection (SCFAs and BAs)
Barrea et al^110^	Participants (n = 137)	—	TMAO was positively correlated with BMI, and specific cut-off of TMAO might help in identifying subjects at high risk of NAFLD	Italy	Targeted metabolomic detection (TMAO)
Zhao et al^111^	HCs (n = 494) Fatty liver disease (n = 273)	—	TMAVA levels were increased in liver steatosis patients	China	Targeted metabolomic detection (TMAVA)
Sehgal et al^112^	HCs (n = 79) Simple Steatosis (n = 49) NASH(n = 45)	Biopsy	In nondiabetic individuals, circulating IPA levels were lower in patients with hepatic fibrosis than in those without	Finland	Nontargeted and targeted metabolomic detection (IPA)
Yu JS et al^113^	HCs (n = 22) NAFLD (n = 25)	Serological models	In the patient group, IPA, IAA, and SCFAs were marginally reduced compared to HCs, while BAs presented elevated levels	Korea	targeted metabolomic detection (SCFAs, BAs, and indoles)

Abbreviations: BA, bile acids; BMI, body mass index; CAP, Controlled attenuation parameter; HCs, healthy controls; IAA, indole-3-acetic acid; IPA, indole-3-propionic acid; MRI-PDFF, MRI-proton density fat fraction; MRE, Magnetic resonance elastography; SCFA, short-chain fatty acids; TMAO, trimethylamine N-oxide; TMAVA, N,N,N-trimethyl-5-aminopentanoic acid.

#### Bile acids (BAs)

Primary bile acids (BAs and goose deoxycholic acid) are synthesized in the liver and then secreted into the duodenum with bile. The intestinal microflora plays an important role in maintaining normal BA metabolism by converting primary BAs to various secondary BAs through processes such as degradation, deoxidation, oxidation, esterification, and desulfation.^114^ There is growing evidence that BAs not only emulsify fats and promote nutrient absorption but also act as signaling molecules to activate farnesylate receptors and bile acid G protein-coupled receptor 5, which play important roles in glucose, lipid, and energy metabolism.^115^,^116^,^117^ Many of these signaling pathways are also thought to be involved in the development of NAFLD as well as in the disease process. In a metabolomics study, patients with NAFLD had higher concentrations of BAs than healthy controls, and the primary BAs GCDCA and GCA, and the secondary bile acids 7-Keto-DCA and GUDCA, bound to glycine, were associated with more severe liver inflammation and liver fibrosis.^107^ A study by Puri P^108^ et al also demonstrated that the composition of BAs changed significantly in patients with NAFLD and that specific changes were associated with the development and severity of steatohepatitis. Chen J et al^106^determined BA profiles in serum and liver tissues by ultra-performance liquid chromatography-tandem mass spectrometry and found that from healthy controls to patients who are non-NASH to patients with NASH, the ratio of conjugated chenodeoxycholic to muricholic acids gradually increased and positively correlated with the degree of steatosis and fibrosis in NASH.

#### Short-chain fatty acids (SCFAs)

Short-chain fatty acids (SCFAs), including formate, acetate, propionate, butyrate, and lactate, are primarily generated by the fermentation of undigested carbohydrates by gut microbiota.^118^ SCFAs play an important role in maintaining the integrity of the intestinal barrier by regulating intestinal luminal pH, secreting mucus, and providing energy for endothelial cells.^119^ Studies have shown that SCFA also have a protective effect against inflammatory bowel disease, type 1 diabetes, and NAFLD.^120^ Therefore, SCFAs could be a potential therapeutic approach to mitigate NASH. For example, the administration of sodium acetate, sodium propionate, or sodium butyrate to methionine- and choline-deficient mice attenuates methionine- and choline-deficient-induced steatosis and inflammation.^121^ Moreover, the intervention of high-fat-fed mice with butyrate inhibits the expression of pro-inflammatory factors IL-1β, IL-6, and MCP1/CCL2 in the liver and toll-like receptors 4 in adipose tissue.^122^ However, elevated levels of SCFAs may also contribute to NAFLD disease progression by maintaining a low-grade inflammatory process, affecting circulating immune cells and peripheral target organs such as the liver.^88^

#### Trimethylamine (TMA) metabolites

Dietary nutrients such as choline and carnitine are broken down by intestinal microorganisms to produce TMA, which is converted to TMAO in the liver by flavin-containing monooxygenases 1 and 3.^123^ TMAO is of wide interest primarily for its role in processes including thrombosis, fat accumulation, and vascular calcification.^124^,^125^ Moreover, increased TMAO levels are linked to an augmented risk of significant adverse cardiovascular events, such as myocardial infarction, sudden death, and stroke.^125^ TMAO can serve as a predictive biomarker for cardiovascular disease, and recently, it has emerged as a candidate risk factor for NAFLD/metabolic-associated fatty liver disease.^125^,^126^,^127^,^128^,^129^ A prospective study including 5292 subjects showed that high TMAO concentrations were associated with increased all-cause mortality in NAFLD.^130^ TMAO may exacerbate hepatic steatosis by regulating BA metabolism. In a case-control study, serum TMAO levels were positively correlated with BA levels in patients with NAFLD, and in a mouse model, TMAO administration impaired liver function and increased hepatic fat deposition.^131^ The effect of TMAO on hepatic steatosis may also be related to endoplasmic reticulum stress. At physiologically relevant concentrations, TMAO binds to endoplasmic reticulum stress enzymes and then selectively activates the unfolded protein response.^132^ Unfolded protein response causes hepatic steatosis, induces apoptosis and inflammation, and is closely associated with the progression of many liver diseases, including NAFLD.^133^ Recently, Zhao M et al^111^ discovered a new microbiota-derived metabolites, N,N,N-trimethyl-5-aminopentanoic acid, may associated with NAFLD. Through nontargeted liquid chromatography-tandem mass spectrometry analysis of plasma samples from patients with hepatic steatosis and healthy controls, the researchers found that plasma N,N,N-trimethyl-5-aminopentanoic acid levels were significantly elevated in patients with hepatic steatosis. Moreover, N,N,N-trimethyl-5-aminopentanoic acid was found to reduce hepatocyte mitochondrial and carnitine synthesis and aggravate fatty liver in mice experiments.

#### Tryptophan metabolites

Tryptophan is an indispensable amino acid that is mostly absorbed by the intestinal epithelium from dietary sources. Over 90% of tryptophan is catabolized via the Kynurenine pathway, while a small fraction (1%–2%) is transformed into 5-hydroxytryptamine.^134^ Unabsorbed tryptophan is metabolized by gut microbiota to indole and its derivatives, mainly including indole-3-aldehyde, indole-3-acetic acid, and indole-3-propionic acid, which play a beneficial role in the development of NAFLD.^134^ Previous studies have suggested a negative correlation between indole levels and liver fat content, with obese individuals exhibiting lower indole levels and higher liver fat content. Furthermore, indole and its derivatives have been shown to possess anti-inflammatory properties and can ameliorate liver steatosis and fibrosis in both in vitro and in vivo models.^135^

### Correlations between metabolites and certain bacterial species

Based on existing research exploring the correlations between gut microbiota abundance and metabolite concentrations in patients with NAFLD, it has been observed that particular bacterial species are associated with specific metabolites (Table 3). In patients with NAFLD displaying elevated liver enzyme ([aspartate aminotransferase] or [ALT] ≥ 50 IU/L), Oscillospiraceae share a significant positive correlation with urocanic acid and 3-hydroxyanthranilic acid, one of the L-tryptophan metabolites.^113^ Another prospective study observed that, in patients with NAFLD with no/mild fibrosis, fecal DCA levels showed a positive correlation with the abundance of Lachnospiraceae and a negative correlation with Bacteroidaceae, whereas serum GCA was positively correlated with Lachnospiraceae and inversely correlated with Bacteroidaceae and Rickenellaceae^137^. It was also found that nonobese NAFLD subjects with higher fibrosis severity presented elevated primary bile acid level, which was associated with increased abundances of Megamonas spp. and decreased abundance of R. bromii, Faecalibacterium prausnitzii, and Roseburia intestinalis.^109^ In addition, a negative correlation between 2-butanone and Ruminococcus and Coprococcus in NAFL individuals,^138^ as well as a connection between the decrease of serum taurocholic acid concentration and Erysipelotrichaceae_UCG-003 have been uncovered in recent years.^104^ Therefore, the association between specific bacterial species and particular gut microbiota-derived metabolites suggests that gut microbiome signatures and metabolic profiles can be used jointly as noninvasive disease biomarkers or applied to construct diagnostic models for the diagnosis or staging of NAFLD.

**TABLE 3:** Correlations between specific bacterial species and certain metabolites and functions of metabolites in NAFLD progression

Substance	Associated gut microbiota	Function
BAs: CA, CDCA, DCA, LCA, and UDCA^136^	Clostridium, Bacteroides, Lactobacillus, Bifidobacterium, Listeria, Faecalibacterium prausnitzii, E. coli, Ruminococcus, Lachnospira, Rickenellaceae, Roseburia, et al	Promote lipid absorption, regulate liver inflammation and glucose metabolism, maintain energy metabolism balance
SCFAs: formate acetate, propionate, butyrate and lactate^88^,^118^	Akkermansia municiphilla, Ruminococcus bromii, Faecalibacterium prausnitzii, Eubacterium rectale, Eubacterium hallii, et al	Maintain gut barrier integrity, regulate immune function and inflammation
TMA^35^,^111^: TMAO, TMAVA	Mainly Firmicutes and Proteobacteria phylum, Bacteroidetes do not produce TMA	Regulate bile acid metabolism; aggravate liver steatosis
Tryptophan metabolites: Indole, IAld, IAA, and IPA^35^	Prevotella, Bacteroides, Fusobacterium, Escherichia, Oscillospira et al	Improve liver inflammation and glucose metabolism

Abbreviations: BA, bile acid; CA, cholic acid; CDCA, chenodeoxycholic acid; DCA, deoxycholic acid; IAA, indole-3-acetic acid; IA1d, indole-3-aldehyde; IPA, indole-3-propionic acid; LCA, lithocholic acid; SCFA, short-chain fatty acids; TMA, trimethylamine; TMAO, trimethylamine N-oxide; TMAVA: N,N,N-trimethyl-5-aminopentanoic acid; UDCA, ursodeoxycholic.

## MICROBIOME-TARGETED THERAPIES OF NAFLD

At present, there are no approved drug therapies for the treatment of NAFLD, with lifestyle changes such as diet and exercise being the primary means of intervention.^139^ However, with a growing understanding of the role of gut microbiota and its metabolites in the development and progression of NAFLD, there is a growing interest in microbiome-targeted therapies for its management. Microbiome-targeted therapies encompass several categories, including probiotics, synbiotics, antibiotics, and fecal microbiota transplantation (FMT).^140^

The probiotics used in clinical trials are mainly Lactobacilli, Streptococci, and Bifidobacteria. Synbiotics are a combination of probiotics and prebiotics, in which prebiotics are dietary fibers that can be fermented by bacteria to produce health benefits.^140^,^141^ Most studies support the positive role of prebiotics/synthetic in the treatment of NAFLD. A meta-analysis suggested that probiotics/synbiotics may have beneficial effects on reducing levels of ALT and improving hepatic steatosis.^140^ However, some inconsistent results have also been seen. A recent randomized controlled trial conducted in the United Kingdom showed that liver fat content and markers of liver fibrosis were not reduced in patients with NAFLD after 1 year of synbiotic administration.^142^ In another clinical trial, probiotic administration did not result in significant clinical improvement in patients with NAFLD; however, probiotics appeared to have the ability to protect the intestinal mucosa in the small intestinal microenvironment.^143^ Certain antibiotics have been utilized for cirrhosis treatment, but further researches are needed on their role in the treatment of NAFLD.^144^ While short-term antibiotic usage can reduce endotoxin and transaminase levels, prolonged usage can lead to bacterial resistance and dysbiosis of the gut microbiota.^144^ FMT is a method of transplanting feces from a healthy donor to a patient to improve the composition of the gut microbiota. Except for being safe for patients, FMT possesses a superior effect than probiotics and synbiotics on the maintenance of intestinal ecology by supplying a variety of commensal bacteria.^22^,^145^ Although current researches have conflicting results regarding the ability of FMT to reduce fat accumulation and insulin resistance, it has the potential to restore gut microbiota diversity and decrease intestinal permeability in patients with NAFLD.^145^

As mentioned above, microbiome-targeted therapies may have the potential to complement the treatment of patients with NAFLD. However, these therapies are not yet formally employed in clinical practice and require further investigation to determine their effectiveness.

## CONCLUSIONS AND FUTURE DIRECTIONS

In this review, we elucidate that gut dysbiosis can be involved in the pathogenesis and progression of NAFLD by eliciting immune dysregulation and metabolism disorder in the host. On top of that, we present a comprehensive overview of the commonly acknowledged gut microbiome signatures and the key metabolic pathways in NAFLD that have been identified by microbiome analyses or metabolomics approaches, with a focus on correlations between specific bacterial species and certain gut microbiota-derived metabolites in patients with NAFLD. In particular, we proposed that BAs have the highest association with gut microbiota, such as Bacteroidaceae, Lachnospira, and Faecalibacterium; Additionally, the abundance of Ruminococcus bromii is closely related to some subclasses of BAs and SCFCs. These already uncovered links indicate that gut microbiome signatures and metabolic profiles have the potential as noninvasive biomarkers for the diagnosis or staging of NAFLD, and hold promise for the development of diagnostic models. Despite the promising prospects of the potential biomarkers, few of them have been validated since changes in microbial abundance and metabolite contents are inconsistent among studies, which largely hinders the advancement and qualification of biomarkers for clinical use in NAFLD. This discrepancy may be attributed to differences in assay platforms and methodologies as different sequencing and metabolomics tools vary in accuracy, sensitivity, and so on. Another possible explanation is that the composition of gut microbiota varies among recruited subjects, leading to a lack of reproduced findings in heterogeneous cohorts.

16S rRNA sequencing and MS are currently the first-line methods to study the gut microbiota and its metabolites. However, 16S rRNA sequencing has limitations in identifying individual species and strains due to the high sequence identity among bacterial families in the hypervariable region. In addition, different studies on the selection of amplification region, primer design, and selection of analysis process will lead to differences in results, thus affecting the comparability of studies. Given the variability in the quantitative changes and roles of different strains within the same genus in NAFLD, it is necessary to conduct studies at the species level. mNGS can identify microorganisms down to the species level or even strain level and also enables in-depth studies at the genetic and functional levels, such as the determination of virulence factors and drug resistance genes, which help provide clues to the mechanistic studies of gut microbiota alteration and NAFLD. In addition, shotgun sequencing features sequencing of all DNA in a sample, which ensures that fungi, archaea, and viruses in the gut microbiome can also be detected and analyzed. Therefore, transitioning from 16S rRNA sequencing to metagenomic analysis may reveal a more profound relationship between the disturbance of mycobiome composition and NAFLD. The primary challenge in MS-based metabolomics is the lack of comprehensive, well-annotated public databases. Incomplete databases make it difficult to effectively interpret results and accurately quantify small molecules due to variations in mass spectra obtained from different experimental conditions and mass spectrometer types. Moreover, the relatively low inter-assay repeatability of metabolomics analysis methodologies results in divergent conclusions from samples collected in different studies. To address these challenges, providing robust bioinformatics support for liquid chromatography-tandem-MS and building self-constructed databases for different types of mass spectrometers are regarded as promising future directions for advancing the field.

Heterogeneity in the selection of study subjects also contributed to the inconsistent results. Previous studies have shown that the composition and function of gut microbiota vary with the host’s age, sex, race, body mass index, diet, disease status, and other factors. Even on the same day, the composition of gut microbiota may change dynamically over time in response to various endogenous and exogenous stimuli. However, current studies generally measure and adjust for only one or few characteristics of the subjects, leading to the overestimation of the correlation between the alterations in the flora and the disease or even conflicting conclusions. Hence, potential confounding factors must be controlled in clinical human studies. Liver biopsy, considered the gold standard for diagnosing NAFLD, is invasive and not suitable for presumed healthy individuals. Therefore, many studies have adopted noninvasive modalities (eg, ultrasound, Fibroscan, and MRI) to classify patients and stage NAFLD, resulting in reduced diagnostic accuracy. Therefore, the establishment of well-characterized clinical cohorts, and the recording and control of confounding factors during the design and data analysis phases of a trial can help to derive the correct association between gut microbiota and NAFLD and enhance comparability among study results.

At present, most studies on gut microbiota and NAFLD only reveal correlations and possible mechanisms, and few reported biomarkers have been validated and utilized in clinical practice. This may be due to the predominantly cross-sectional design of these studies, which not only precludes the establishment of causal relationships between alterations in gut microbiota and metabolites and the onset of fatty liver disease but also leads to the lack of relevant studies using gut microbiota and its metabolites as diagnostic models for NAFLD. Recently, a prospective study by Leung et al^87^ developed a risk prediction model for NAFLD based on the microbiome characteristics of subjects in a prospective cohort, which can be used to predict the risk of NAFLD in healthy subjects over the next 4 years. This study also demonstrated that the gut microbiota composition and function of patients differ from those of healthy subjects even before ultrasound diagnosis of NAFLD, indicating its potential as a noninvasive diagnostic test for NAFLD. Therefore, further longitudinal cohort studies are needed in the future to extend the cross-sectional evidence.

In conclusion, a mounting number of animal experiments and clinical studies have highlighted the key role of gut microbiota and metabolites in the pathogenesis of NAFLD, indicating their potential utility in the diagnosis, staging, and treatment of NAFLD. Nevertheless, the full potential of these entities as noninvasive biomarkers and their clinical applicability remains largely untapped. The establishment of the MASLD nomenclature and the adoption of affirmative criteria, rather than exclusionary ones, offer definitive and unequivocal descriptions. Additionally, the Delphi consensus facilitates more precise classification and diagnosis of metabolic and alcohol associated liver disease (subjects with alcohol intake greater than that allowed for MASLD) and cryptogenic SLD (subjects exhibiting no metabolic parameters and no identifiable known cause) within the broader SLD category.^1^ This advancement addresses the challenges encountered in the design of clinical trials and drug treatments arising from the heterogeneous nature of the NAFLD population. Moreover, well-designed longitudinal and prospective cohort studies are urgently required to overcome the limitations of population variability in demographics and validate potential microbiota-derived biomarkers. Considering the constraints of existing methodological approaches, identifying more specific markers for diagnostic models using advanced computational science and multi-omics analysis combining genomics, metabolomics, and lipidomics could represent an important research direction in the future.
